# PIMMS (Pragmatic Insertional Mutation Mapping System) Laboratory Methodology a Readily Accessible Tool for Identification of Essential Genes in *Streptococcus*

**DOI:** 10.3389/fmicb.2016.01645

**Published:** 2016-10-25

**Authors:** Adam M. Blanchard, Sharon A. Egan, Richard D. Emes, Andrew Warry, James A. Leigh

**Affiliations:** ^1^School of Veterinary Medicine and Science, University of NottinghamSutton Bonington, UK; ^2^Advanced Data Analysis Centre, University of NottinghamSutton Bonington, UK

**Keywords:** mutagenesis, insertion sequencing, essential genome, *Streptococcus*, laboratory protocol

## Abstract

The Pragmatic Insertional Mutation Mapping (PIMMS) laboratory protocol was developed alongside various bioinformatics packages (Blanchard et al., 2015) to enable detection of essential and conditionally essential genes in *Streptococcus* and related bacteria. This extended the methodology commonly used to locate insertional mutations in individual mutants to the analysis of mutations in populations of bacteria. In *Streptococcus uberis*, a pyogenic *Streptococcus* associated with intramammary infection and mastitis in ruminants, the mutagen pGhost9:ISS1 was shown to integrate across the entire genome. Analysis of >80,000 mutations revealed 196 coding sequences, which were not be mutated and a further 67 where mutation only occurred beyond the 90th percentile of the coding sequence. These sequences showed good concordance with sequences within the database of essential genes and typically matched sequences known to be associated with basic cellular functions. Due to the broad utility of this mutagen and the simplicity of the methodology it is anticipated that PIMMS will be of value to a wide range of laboratories in functional genomic analysis of a wide range of Gram positive bacteria (*Streptococcus, Enterococcus*, and *Lactococcus*) of medical, veterinary, and industrial significance.

## Introduction

Streptococci are significant pathogens of man, animals, aquatic mammals, and fish (Chanter, 1997). Some show a high degree of host and disease specificity whilst others are able to cause a wide array of different pathologies in distinct host targets (Steer et al., 2012). Many streptococcal species (including pathogens) are also able to co-exist in an asymptomatic carriage state with their host (Murphy and Frick, 2013); while others previously considered benign commensals, are now associated with colon cancer and endocarditis in humans (Chadfield et al., 2004; zur Hausen, 2006).

*Streptococcus uberis* is a member of the pyogenic cluster of *Streptococcus*. Although, able to colonize the bovine gut asymptomatically, intramammary infection with this bacterium is one of the most common causes of bovine mastitis worldwide (Bradley et al., 2007); resulting in huge financial losses to the dairy industry and the requirement for large quantities of therapeutic antibiotics (Pol and Ruegg, 2007). *S. uberis* is amenable to insertional mutagenesis with the temperature sensitive mutagen, pGhost9:ISS1 (Ward et al., 2001), which has been used similarly in other species of *Streptococcus, Lactococcus*, and *Enterococcus* to gain insight of the role of individual bacterial sequences (Maguin et al., 1996; Spellerberg et al., 1999; Biswas and Biswas, 2011; Baureder and Hederstedt, 2012).

An understanding of the contribution of the entire bacterial genome to biological processes will enable a more comprehensive evaluation of microbial physiology and biochemistry. In doing so, it will be possible to identify bacterial gene products and combinations of gene products responsible for bacterial proliferation and survival against which new therapeutics and preventative disease controlling agents can be developed.

The use of random insertional mutagenesis coupled with high throughput sequencing technologies has enabled identification of essential and conditionally essential genes for many pathogenic bacteria. Various protocols have been developed to achieve this including; Tn-Seq (van Opijnen et al., 2009), INSeq (Goodman et al., 2009), HITS (Gawronski et al., 2009), and TraDIS (Langridge et al., 2009). These each require a series of complex steps to produce mutants and isolate DNA fragments flanking insertions and in some cases specialized sequencing procedures are required to generate the final data set. Various bioinformatic approaches and predictive modeling strategies have been used to analyze the vast amounts of data produced from such protocols (Zomer et al., 2012; Chao et al., 2013; Pritchard et al., 2014; Blanchard et al., 2015).

The use of inverse PCR of re-circularized restriction fragments to amplify sequences flanking insertions has been used for determination of the sites of individual mutations in various bacterial species including; *Pseudomonas abietaniphila* (Martin and Mohn, 1999), *Mycoplasma genitalium* (Hutchison et al., 1999), *Xanthomonas albilineans* (Huang et al., 2000), *Helicobacter pylori* (Salama et al., 2004), and *S. uberis* (Ward et al., 2001). However, methods that combine this commonly used strategy with high throughput technologies, to enable simultaneous analysis of bacterial mutant populations, have not been developed. The wide applicability of pGh9:ISS1 as a mutagen in *Streptococcus* (and related bacterial species) makes this an attractive target around which such technology may be produced.

In this communication, we describe the development and application of a simple, accessible laboratory protocol Pragmatic Insertional Mutation Mapping (PIMMS laboratory protocol), with wide applicability to any bacterial species mutated with pGh9:ISS1, using an existing bank of *S. uberis* mutants (Ward et al., 2001).

## Methods

### Generation of Bacterial Mutant Pools

A culture of the bovine isolate of *S. uberis* 0140J that had been mutagenized (Ward et al., 2001) with the thermosensitive plasmid containing the insertion sequence element S1 (pGh9:ISS1) and stored at -80°C was used throughout this study. The viability and frequency of pGh9:ISS1 insertions within the culture were assessed by serial dilution plate counts on Todd-Hewitt agar (THA; Oxoid, UK) in the presence and absence of erythromycin (Ery; 1 μg/ml; Sigma Aldrich, UK). Total counts in the presence/absence of Ery were used to calculate the total number and the proportion of mutant bacteria within the culture.

Subsequently, a sample of the mutagenised culture, diluted appropriately, was plated and grown to single colonies on THA containing Ery (1 μg/ml). Ten pools each containing approximately 10^4^ colonies were scraped from plates into phosphate buffered saline (PBS; Gibco, ThermoFisher, UK) and resulting bacterial suspension collected by centrifugation (8000 × *g*, for 10 min) washed (three times) in PBS, and finally suspended in pyrogen free saline (Sigma Aldrich, UK) containing 50% (v/v) glycerol. These pools were stored in aliquots and frozen at -80°C.

### DNA Extraction

Chromosomal DNA was extracted from bacteria according to the method (Hill and Leigh, 1989). The final DNA sample was obtained by centrifugation (12, 000 × *g* for 5 min), and following removal of the supernatant was allowed to air dry at ambient temperature before being suspended in TE buffer containing 20 μg/ml RNAse A. DNA was quantified using the Qubit dsDNA Broad Range Fluorometric Assay kit (Life Sciences, UK), according to manufacturer’s instructions.

### Preparation of DNA for Inverse PCR Reaction

Restriction digests of DNA using *HindIII* and *EcoR1* were performed by the addition of 10 units of restriction enzyme to a total reaction volume of 50 μl using 1 μg of DNA and incubated for 1 h at 37°C. The reaction was then heat inactivated at 80°C for 20 min. The digested DNA was purified using PCR cleanup kit (Machery and Nagel, USA) and eluted using 30 μl of pre-heated (70°C) elution buffer.

Approximately 6 μg of genomic DNA was suspended in 200 μl TE buffer and fragmented to an average size of 3 kb using Covaris Adaptive Focused Acoustics (Covaris, Inc., USA) according to the manufacturer’s protocol. The fragmented DNA was purified using Agencourt SPRI beads (Beckman Coulter, UK) according to the manufacturer’s protocol; briefly 1.8x volume of beads was added to the DNA, mixed by pipetting and allowed to incubate at room temperature for 5 min. The beads containing DNA were separated from the supernatant using a magnetic stand and the supernatant aspirated. Beads were washed twice with high purity 70% ethanol, and the DNA eluted in molecular biology grade water (Fisher Scientific, UK). The size distribution of DNA fragments was quantified using an Agilent Bioanalyser in line with the standard protocols. Fragmented DNA was blunt end repaired using the NEBNext End Repair module (New England Biolabs, Inc., USA), purified with 1.8x SPRI beads (as previously described) and resuspended in 50 μl molecular biology grade water.

The end-repaired or restriction digested DNA (1 μg) was suspended in 750 μl ligase buffer in the presence of 1000U T4 ligase (New England Biolabs, Inc., USA); and incubated at 22°C overnight. The DNA was purified and concentrated using a PCR clean-up kit (Machery and Nagel, USA) and eluted using 30 μl of pre-heated (70°C) elution buffer.

### Inverse PCR

An inverse PCR was conducted to enrich the sequence flanking the ISS1 element. In a 50 μl reaction volume, 100 ng of re-circularized DNA was used as template with 2 mM dNTPs and 10 pmol of each primer (P082 5′-CCAACAGCGACAATAATCACATC-3′ and P064 5′-AGAACCGAAGAATTCGAACGCTC-3′). The reaction was incubated for 5 min at 98°C before the addition of 1 U of Phusion High fidelity DNA polymerase (New England Biolabs, Inc., USA) to initiate the reaction (denature of 98°C for 2 min followed by 35 cycles of 98°C for 10 s, 63°C for 30 s, 72°C for 1 min with a final extension of 8 min at 72°C). The PCR products were isolated using 1.8-volumes of Agencourt SPRI beads (Beckman and Coulter, UK) as previously described and suspended in 30 μl of Molecular biology water (Fisher Scientific, UK).

### Nucleotide Sequencing

The purified PCR products were fragmented to 550 bp using Covaris Adaptive Focused Acoustics (Covaris, Inc., USA) following the manufacturer’s directions. This size distribution of DNA fragments was estimated using an Agilent Bioanalyser 2100 using the DNA7500 kit (Agilent Technologies, USA) in line with the standard protocols. The samples were prepared for sequencing on the Illumina MiSeq platform at 2 × 250 bp reads using the Illumina TruSeq Nano library preparation kit (Illumina, Inc., USA).

### Analysis of Data

Raw FASTQ files containing all fragment reads accompanied by quality scores and read identifiers from the sequence run generated by the MiSeq were analyzed using freely available software on a Linux system. The PIMMS pipeline (Blanchard et al., 2015) was used to process the reads and map them to the *S. uberis* 0140J reference genome [accession number AM946015 (Ward et al., 2009)]. Briefly, each sequence read was assessed for the presence of the terminal portion of ISS1 [accession number (of pGh9:ISS1) EU223008.1] and once identified, the remaining sequence was analyzed for quality. To ensure high quality mapping to the bacterial genome, each read was required to provide a Phred score of >30 (each base has a 99.9% confidence level) and adhere to a minimum (21 bp) length restriction, to ensure an unequivocal alignment. Only reads that reached these satisfactory quality and length requirements were mapped against the *S. uberis* genome. The mapping parameters were set to restrict any mismatch, to maintain high alignment accuracy and minimizing the likelihood of sequence ambiguity.

## Results

### Development of the PIMMS Protocol

The quality of the sequence data generated from each of the enriched samples was comparable to that obtained following direct sequencing of gDNA; all sequence data had an average Phred score of >35 and no single base had a Phred score of lower than 31 (**Table 1**).

**Table 1:** Analysis of sequences by inverse PCR obtained from gDNA and preparations enriched for DNA flanking pGh9:IS*S1* insertions.

	DNA fragmentation method			
Measured parameter	*Eco*R1	*Hin*dIII	Covaris	None^1^
Total sequence reads (TSRs)	3,848,211	3,294,706	2,977,706	3,377,794
Average Phred score	37.26	37.31	37.48	36.75
Minimum base Phred score	33.33	33.6	32.23	31.59
Maximum base Phred score	38.48	38.48	38.35	38.62
Matched reads (%)^2^	2,400,817 (62.39)	2,075,014 (62.98)	1,380,699 (40.88)	5,817 (0.20)
Mapped reads (%)^3^	937,081 (39.03)	613,441 (29.56)	341,562 (24.74)	1,439 (24.74)
Unique insertion points (UIP)	5,835	9,657	21,834	721
Average read depth (TSR/UIP)	160.6	63.5	15.6	1.9
Average inter-insertion distance (bp)	89	72	41	1054

*Data on each CDS is located in **Supplementary Table S1**. ^1^Not subjected to Inverse PCR enrichment gDNA only. ^2^Number of sequence reads containing 23 bp of either terminus of IS*S1.*^3^Number of Matched sequence reads containing >20 bp of unambiguous chromosomal sequence adjacent to IS*S1* sequence.*

Sequence data was analyzed and mapped back to the original genome using the PIMMS bioinformatic pipeline (Blanchard et al., 2015). Comparative analysis indicated that the number of sequence reads that included 23 bp from either terminus of the insertion sequence (Matched Reads; **Table 1**) was similar for the *EcoR1* and *HindIII* digested samples (62.3% and 62.9%, respectively). Proportionally fewer sequence reads (40.8%) produced from acoustically fragmented samples contained the equivalent ISS1 sequences and only very few (0.2%) of the sequence reads obtained directly from the untreated gDNA sample contained either terminus of the insertion sequence (**Table 1**).

A bioinformatic pipeline (Blanchard et al., 2015) was used to map the sequence directly adjacent to the IS*S1* terminus to the *S. uberis* reference genome (accession number AM946015; Ward et al., 2001). This revealed that between 39 and 25% of the matched reads mapped unambiguously to the source genome. However, the number of unique matches (unique mutations) showed that the libraries produced from endonuclease digestion were markedly less diverse than that generated by random acoustic shearing of gDNA (**Table 1**).

**FIGURE 1:**
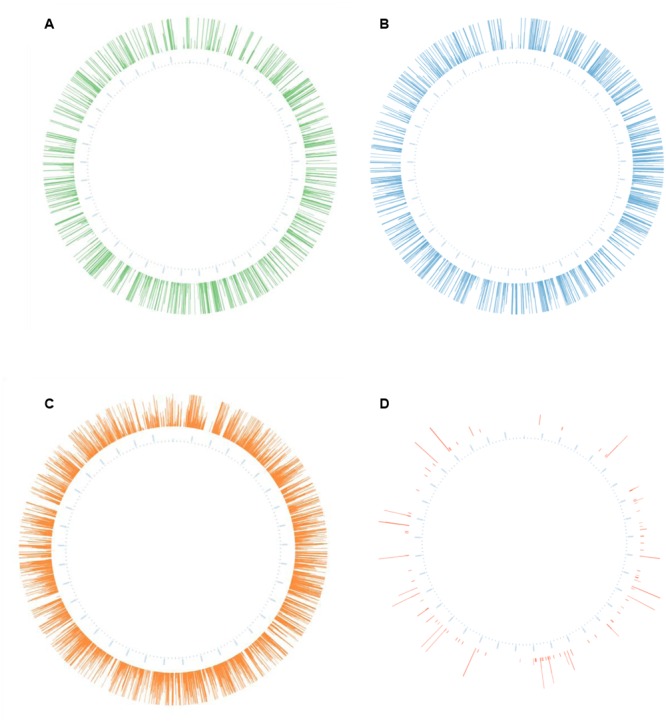
**Representation of unique insertions following analysis of different sequencing libraries.** The genomic coordinates of each unique insertion were used to generate corresponding Circos plots (Krzywinski et al., 2009) of mutations in the genome of *Streptococcus uberis*. The height of each line represents mutation read depth (set to a maximum of fifty reads and a minimum of three). The libraries were generated from **(A)** *EcoR1* digested DNA; **(B)** *HindIII* digested DNA; **(C)** Acoustically fragmented DNA; or **(D)** Unenriched gDNA.

In each case, these libraries were enriched for flanking sequences (matched reads) compared to that produced from non-enriched gDNA; the *EcoR1*-based library was enriched 651-fold and those generated using *HindIII* and acoustically (randomly) sheared DNA were enriched 426 and 237 fold, respectively (**Table 1**). In all cases, locations of mutations were shown to be dispersed around the entire bacterial chromosome (**Figure 1**).

### Comparison of the Sample Production Methods on Insertion Discovery

**FIGURE 2:**
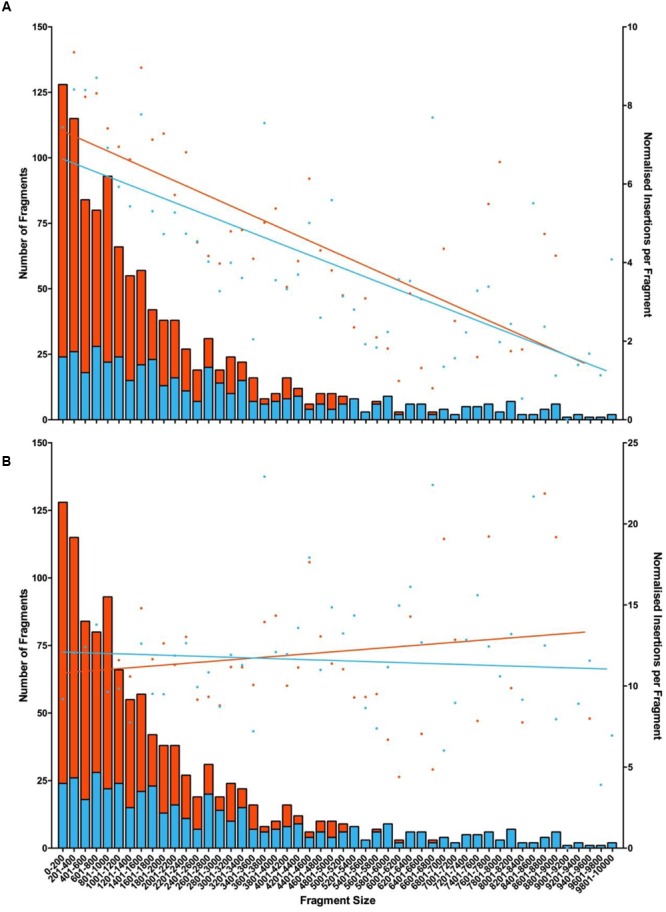
**Mapping insertions to restriction fragments following fragmentation with the corresponding endonuclease or by random acoustic shearing.** Fragment length produced by either endonuclease was calculated. The number of insertions located to each fragment was determined and a linear regression of normalized insertion (NIM; Blanchard et al., 2015) was used to show any trends. Data represents NIM (dots right axis) mapped to fragments produced with endonuclease (left axis, orange bars = *HindIII*; blue bars = *EcoRI*). **(A)** NIM from corresponding endonuclease generated sample or **(B)** or acoustic sheared sample. *R*-values chart **(A)** HindIII (0.5476) and EcoR1 (0.5283); chart **(B)** HindIII (0.0255) and EcoR1 (0.0057).

As fewer unique insertion points were detected in the two libraries generated from inverse PCR products of endonuclease digested gDNA than in that generated by randomly shearing DNA prior to re-circularisation, it can be assumed that endonuclease fragmentation with either *HindIII* or *EcoRI* introduced bias in the process. This was assessed using *in silico* digestion of the *S. uberis* genome and the association of all insertions found within boundaries of each pair of restriction sites (**Figure 2**). This demonstrated the tendency for smaller restriction fragments to yield insertion data (**Figure 2A**); mapping insertions from the randomly sheared samples was largely independent of the length of the theoretical restriction fragments (**Figure 2B**).

**FIGURE 3:**
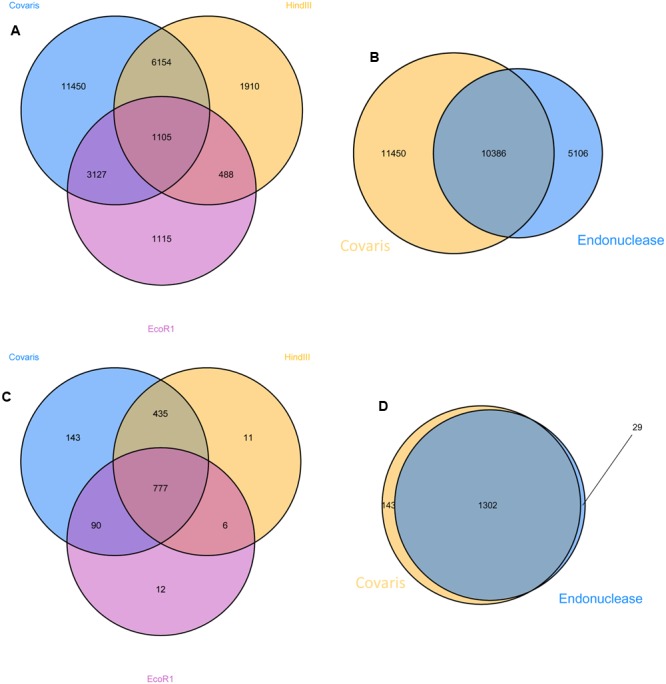
**Comparison of mutations detected in samples prepared by endonuclease digestion and random acoustic fragmentation (Covaris).** Venn diagrams to demonstrate the overlap of mapped sequences detected from sample prepared using different procedures; **(A)** indicates the number of unique insertions using each procedure; **(B)** indicates the number of unique insertions using both endonuclease generated preparations compared to that generated by random acoustic shearing (Covaris); **(C,D)** as **(A,B)**, respectively, using mutated coding sequence as the unit of definition.

Correspondence of the insertion data from the three enrichment protocols was investigated at the level of unique insertion discovery and at the resolution of coding sequence disruption (**Figure 3**). A high proportion (87.5%) were discovered in the library originating from randomly sheared gDNA, whereas considerably fewer (21.7 and 35.8%) were identified in the libraries produced by digestion with *EcoR1* and *HindIII*, respectively. However, more than half the insertions (57.5%) were detected by combining data obtained from both libraries produced from endonuclease digested gDNA. Despite the clear superiority of using randomly sheared gDNA as the starting material in this process, the number of mutated coding sequences detected in libraries generated with randomly sheared or endonuclease fragmented gDNA were similar. Of 1474 mutated coding sequences (CDS), a very high proportion (98%) was identified using the randomly sheared sample library and 83.4 and 60.0% were identified using the *HindIII* and *EcoRI* generated samples, respectively. Cumulatively, the endonuclease generated libraries yielded insertion data in approximately 90% of the total mutated CDS identified in the study.

### The Application of PIMMS for Identification of Genes Essential for Bacterial Growth

Mutated cultures were plated on to solid media containing erythromycin and harvested in saline and stored at -80°C in the presence of glycerol to produce 10 amplified pools of mutants these were processed as previously described using acoustically sheared gDNA as the starting material (**Table 2**).

**Table 2:** Evaluation of sequences generated from 10 pools of *Streptococcus uberis* mutants.

Pool number	Total number of sequence reads	Matched reads (%)^1^	Mapped reads (%)^2^	Unique insertionswith ≥3 occurrences^3^	Average read depth	Number of CDSlacking insertions
1	8,953,812	4,437,287 (49.6)	2,178,162 (49)	10,647	204	574
2	7,861,879	2,426,829 (30.9)	1,071,066 (44)	13,669	78	473
3	8,277,653	4,183,217 (50.5)	1,335,140 (32)	15,287	87	450
4	8,115,818	3,003,635 (37)	1,153,172 (38)	15,611	73	446
5	8,268,603	3,672,825 (44.4)	176,272 (4.8)	6,004	28	648
6	6,761,822	1,942,516 (28.7)	797,029 (41)	7,356	108	695
7	8,636,714	2,446,844 (28.3)	836,874 (34)	8,856	94	596
8	6,478,983	1,795,475 (27.7)	881,896 (49)	9,719	90	618
9	7,266,178	2,187,573 (30.1)	961,217 (43.9)	8,780	109	650
10	8,526,696	4,220,350 (49.5)	2,143,901 (50.7)	19,561	109	375
Pooled^4^	**79,148,158**	**30,316,551 (38.3)**	**11,534,729 (38)**	**80,617**	**182**	**196**

*Data on each CDS is located in **Supplementary Table S1**. ^1^Sequence reads which contain 23 bp of IS*S1* terminal sequences. ^2^Matched sequence reads in which 21–50 bases adjacent to the ISS1 terminus have also been mapped to the reference genome [accession number AM946015 (Ward et al., 2009)]. ^3^The number of unique flanking sequences identified within the mapped reads with >3 occurrences (Goodman et al., 2009). ^4^Total unique mutations mapped in all pools.*

Analysis of the combined sequence data from the mutant pools (approximately 10^5^ individual mutant colonies) identified 80,617 unique insertion points; each mutated CDS having an average of 31 unique insertions. Analysis of the locations of all unique insertions within the genome identified 196 CDS where no insertion event was identified (**Table 3**) and a further 67 CDS where mutations were only detected in the last 10th percentile of the CDS; termed truncated genes (**Table 4**). Essential and truncated sequences were classified using RAST (Rapid Annotation using Subsystem Technology; Overbeek et al., 2014). The majority of non-mutated sequences were associated with transcription and translation and other basic cellular functions including those involved in catabolism and cell cycle (**Table 3**). The truncated sequences were dominated by genes associated with the synthesis of ribosomal proteins (**Table 4**).

**Table 3:** Rapid Annotation using Subsystem Technology (RAST) classification of *S. uberis* CDS containing no insertions.

RAST category	Count	Gene
Amino acids and derivatives	2	*aspS, alr*
Carbohydrates	12	*fba, pgk, plr, gpmA, acoL, pfk, pdhC, pdhB, ptsH, gapN, pgi, gpsA*
Cell division and cell cycle	8	*ftsA recU, ftsL, ftsZ*, SUB1127, 1285, 1404, 1092
Cell wall and capsule	14	*glr, dltC, murF, ddlA, murG, rmlB, murE, pbpX, glmS, rmlA, rlmC*, SUB0010, 0696, 0697
Cofactors, vitamins, prosthetic groups	4	*ppnK, dpfB, birA*, SUB0641
DNA metabolism	11	*mecA, dnaI, dnaG, hlpA, ssb, dnaC, parE, dnaH, plsX, xseB*, SUB1777
Fatty acids, lipids, and isoprenoids	18	*aacA, aacD, aacC, fabZ, uppS, dgk, mvaD, fni, fabD, fabK, acpP, fabH, a, mvaS, fabE, fabF*, SUB1015, 1501
Hypothetical proteins	9	*veg*, SUB0149, 0332, 0388, 0726, 0930, 1286, 1547, 1619
Iron acquisition and metabolism	0	–
Membrane transport	6	SUB0581, 1004, 1158, 1413, 1799, 1853
Miscellaneous	11	*prsA1, secG, ftsE*, SUB0223, 0382, 0399, 1093, 1472, 1620, 1732, 1775
Nucleosides and nucleotides	6	*prsA2, tmk, nrdH, adk, ybeY* SUB1225
Phosphorus metabolism	0	–
Protein metabolism	58	*alaS, engA, engC, fus, gatA, gatB, gltX, groES, infA, infB, infC, pheS, prfB, rimM, rplB, rplD, rplD, rplE, rplF, rplJ, rplK, rplK, rplN, rplO, rplP, rplR, rplS, rplU, rplV, rplW, rplX, rpmA, rpmB, rpmC, rpmD, rpme, rpmF, rpml, rpmJ, rpsB, rspC, rpsD, rpsE, rpsF, rpsG, rpsH, rpsI, rpsJ, rpsK, rpsL, rpsM, rpsN, rpsO, rpsQ, rpsS, rpsU, tufA*, SUB1008, 1732A
Regulation and cell signaling	1	*hisS*
Respiration	7	*atpB, atpD, atpE, atpF, atpA, atpG, atpH*
RNA metabolism	13	*leuS, asnS, glyS, proS, rnpA, era, trmD*, SUB0849, 1470, 1467, 1616, 1618, 1847
Stress response	3	*dnaJ, grpE*, SUB0009
Virulence, disease and defense	4	*vicR*, SUB0502, 0505, 0506
Total	196	

**Table 4:** Rapid Annotation using Subsystem Technology classification of *S. uberis* CDS containing insertions only within the last 10% of the CDS.

RAST category	Count	Gene
Amino acids and derivatives	2	*cysE, mtnN*
Carbohydrates	2	*ptsK, tpi*
Cell division and cell cycle	2	*ftsW, ftsX*
Cell wall and capsule	10	*bacA, gcaD, hasC, mraY, murB, murC, murM, rgpG*, SUB426, 0700
Cofactors, vitamins, prosthetic groups	7	*coaA, coaD, coaC, dyr, metK, nadE*, SUB0356
DNA metabolism	5	*dnaA, dnaN, holB, parC, gyrA*
Fatty acids, lipids, and isoprenoids	5	*mvaA, mvaK2*, SUB253, 0333, 1246
Hypothetical proteins	2	SUB1434, 1834
Iron acquisition and metabolism	0	–
Membrane transport	5	*secA*, SUB0511, 1005, 1852, 1854
Miscellaneous	4	*tyrS*, SUB0019, 0393, 0704
Nucleosides and nucleotides	5	*gmk, pgmA, thiD*, SUB0745, 1227
Phosphorus metabolism	1	*ppaC*
Protein metabolism	11	*argS, efp, prfA, pth, rplL, rplM, rplQ, serS, thrS, trsA*, SUB0345
Regulation and cell signaling	0	–
Respiration	0	–
RNA metabolism	6	*ileS, nusA, rpoD*, SUB0013, 0873, 1479
Stress response	0	–
Virulence, disease and defense	0	–
Total	67	

**FIGURE 4:**
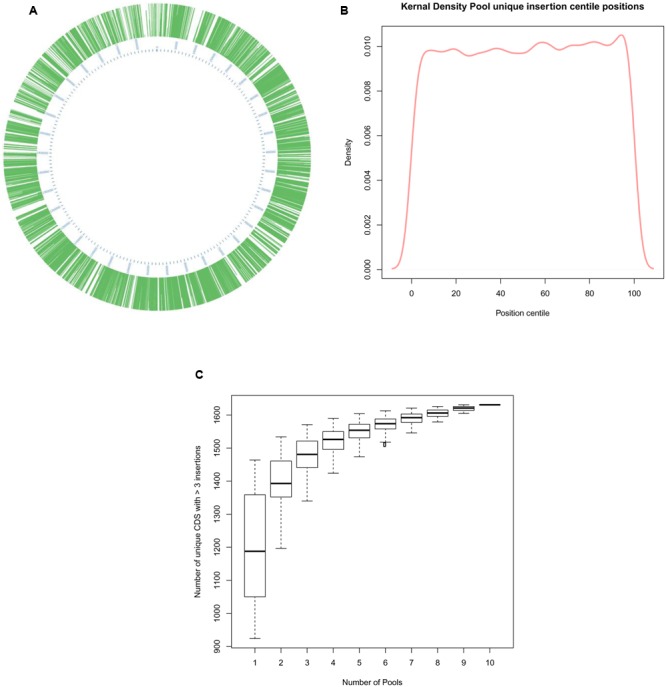
**Analysis of >80,000 unique insertions detected within the genome of *S. uberis* following PIMMS. (A)** Circos graphical representation (Krzywinski et al., 2009) of the distribution of unique insertions identified within the *S. uberis* genome. **(B)** Kernal density plot displaying the proportion of individual mutations identified each centile position of mutated CDS within the *S. uberis* genome. **(C)** Box and whisker plot showing inter quartile range (box), median (line) and range (whiskers) of unique insertions detected following a random permutation test (1000 permutations of 1 million randomly selected sequence reads from each pool were used to calculate mean number of disrupted CDS that may be detected from any 1 of 10, 2 of 10, 3 of 10, etc. to 10 of 10 pools).

Insertion coordinate data was used to determine their location in the genome (**Figure 4**). As previously detected, insertions were dispersed around the entire genome (**Figure 4A**) and a kernel density plot showed insertions typically occurred along the entire length of mutated CDS (**Figure 4B**) The PIMMS counts package (Blanchard et al., 2015) was used to evaluate the density of mutations identified within mutated CDS, the rate of insertion was found to be an average of 140 insertions per kb of CDS within the mutated genome. The genome sequence preceding the insertion point was evaluated to assess for the presence of any insertional motif; none was detected.

To assess the level of redundancy in data acquisition the raw sequence-read data were subjected to a random permutation test using 1000 permutations for each parameter. Initially, samples (1 million sequence reads) randomly selected from each pool was used to calculate the number of mutated CDS detected in each. These data were used to calculate the mean number of mutated CDS detected and the upper and lower quartiles. The process was repeated for any 2 out of 10 pools, any 3 out of 10 pools and so on up to 10 out of 10 pools (**Figure 4C**). This indicated that obtaining 1 million sequence reads from any five pools of mutants was likely to generate >85% of the total informative data relating to CDS requirement gained from sequencing all 10 mutant pools.

Using orthologous genes, the position and frequency of unique insertions were superimposed on to the glycolytic pathway (Kegg pathway identifier:sub00010) using Pathview (Luo and Brouwer, 2013). This revealed that most genes with unique 1:1 orthologs contained no insertions or contained insertion beyond the 90th percentile of their sequence. However, both enolase (sub0655) and pyruvate kinase (sub1000) tolerated mutation starting at the 39th and 46th percentile of their sequences, respectively. Mutations were present at low frequencies (sub0655 = 3.1 and sub1000 = 0.66 unique insertions/kb CDS) and these insertions were not abundant with Normalized Read Scores (NRM; Blanchard et al., 2015) of 16.86 and 0.17, respectively for each CDS; compared to a mean NRM of 539.33 for all (mutated and non-mutated) CDS in the genome. In this pathway, interconversion of glycerate-2P and glycerate-3P can be effected by two distinct enzymes, phosphoglycerate mutase and 2,3-di-phosphoglycerate-dependant phosphoglycerate mutase. Phosphoglycerate mutase has four orthologs in *S. uberis* (sub0594; sub0838, sub0839, and sub1509) all of which showed insertions throughout their sequences at frequencies ranging from 38.7 to 72.6 unique insertions/kb. Whereas, 2,3-di-phosphoglycerate-dependant phosphoglycerate mutase has only a single ortholog (sub1263; *gpmA*), this did not contain insertions.

## Discussion

The laboratory methodology (PIMMS laboratory protocol) described in this communication was an adaption and extension of techniques used previously to map individual mutations generated by the insertional mutagen pGh9:IS*S1* (Ward et al., 2001). By combining high-density random mutagenesis and readily available DNA sequencing protocols, PIMMS laboratory protocol was able to comprehensively generate data to identify mutated sequences in populations of *S. uberis* using the PIMMS bioinformatics packages (Blanchard et al., 2015). The ability to utilize bacteria mutagenized with pGh9:IS*S1* in this manner is a significant advance in the repertoire of tools available for functional genomics of *Streptococcus, Lactococcus*, and *Enterococcus* in which production of banks of random mutants using this mutagen is similarly straight forward (Maguin et al., 1996).

The PIMMS protocols are relatively simple and sequence data was produced using conventional, and thus readily available, library preparation, and sequencing protocols. In line with previous studies using this mutagen (Maguin et al., 1996) no obvious insertion motif was detected; the PIMMS bioinformatic pipeline provided data on base frequencies of insertion positions and whilst a slight bias for AT was seen (28.8% A and 25% T) this is consistent with the AT nucleotide content (63.4%) of *S. uberis*.

Detection of insertions within the non-enriched gDNA sample was in line with that predicted using the formula: (RS)/g = gn where ‘R’ is the number of sequence reads (paired end) for the sample (2,977,706); ‘S’ is the number of bases sequenced per read (500); ‘g’ is the length of the genome (1,852,352 bp); and ‘gn’ equals the number of genome equivalents sequenced; the predicted value for gn was 803. The actual number of unique insertions detected was 721; ∼90% of that predicted.

The efficiency with which the protocol detected IS*S1* junctions using endonuclease-fragmented gDNA was higher than that obtained with randomly sheared gDNA. However, the diversity of the insertions mapped using endonuclease digested samples was reduced compared to those data obtained using acoustically fragmented gDNA. This may be explained by the proximity of the specific restriction enzyme recognition sites and the point of insertion, as we were able to demonstrate that insertions were preferentially detected from shorter restriction fragments in their corresponding endonuclease prepared samples. Furthermore, this effect was abolished when mapping insertions detected using the randomly sheared gDNA sample to these sequences (**Figure 2**). The irregular distribution of such restriction sites within *S. uberis* (this particular strain contains 1086 *HindIII* and 495 *EcoR1* restriction sites) renders these more simple methodologies for gDNA fragmentation less useful in a comprehensive, genome-wide analysis of mutated populations. However, when data was analyzed by CDS mutation, the combined data obtained from both libraries produced by *EcoRI* and *HindIII* could be used to detect 90% of the total mutated CDS, of which 87.5% could be detected using gDNA fragmented with *HindIII* alone (compared to 98% detected in libraries produced from randomly sheared gDNA). Consequently, in the absence of the capability to generate precisely-sized, randomly sheared gDNA and with the application of carefully controlled experimental protocols (Chao et al., 2016), analysis of combinations of sequencing libraries made from inverse PCR products derived from endonuclease digestion of gDNA may be a practical alternative to identify conditionally essential CDS.

The movement toward next generation high-throughput transposon insertion-site sequencing in Streptococci has been shown in studies on *Streptococcus pneumoniae* (van Opijnen et al., 2009), *Streptococcus pyogenes* (Le Breton et al., 2015), and *Streptococcus agalactiae* (Hooven et al., 2016) and these provide a benchmark for the PIMMS protocol. In all cases the Tn-seq protocol was used for detection of the insertion junction sequences. In the initial studies using *S. pneumoniae* a population of 150,000 mutants was used and only 23,875 unique insertions (15.9%) were detected. In the present study, the pGh9:ISS1 mutagen compared favorably; in a population of approximately 115,000 mutants, 80,617 (∼70%) unique mutations were detected. These data further support the assertion that pGh9:ISS1 has neither a transposition bias to a specific insertion motif (Green et al., 2012) nor holds an insertional preference to specific structural features of DNA (Lampe et al., 1998); either of which can lead to pseudo-random insertions, limiting the range and variability of mutations that can be created within a population. In the studies in *S. pyogenes* (Le Breton et al., 2015) and *S. agalactiae* (Hooven et al., 2016) the mutation/mutant ratios were not reported.

Mutagenesis with pGhost9:ISS1 is very straight forward and enables production of mutagenized pools of bacteria that have undergone very little manipulation and/or inter-strain competition. In the current study, the only post-mutagenesis selection of mutants was their ability to survive a short outgrowth period (typically 2.5 h after transfer to the non-permissive temperature; Ward et al., 2001), storage at -80°C and to produce a colony on solid media containing erythromycin. Viable counts of harvested pools indicated that total and erythromycin resistant counts were the same and that approximately 10^7^ cfu of *S. uberis* were obtained per harvested colony; thus each suspension contains an amplified pool of mutated bacteria in which the frequency of mutations approximated to the number of mutant colonies harvested. This enabled pools of mutants to be prepared as a reagent for subsequent repeated use, thus permitting greater cross comparison between studies of different phenotypic selections.

The efficiency with which the PIMMS pipeline identified matched and mapped reads was in line with expectations in consideration of the associated laboratory protocols. The average length of the PCR product from the randomly sheared and re-circularized gDNA template is ∼2 kb; fragmentation of this to 550 bp for sequencing library preparation would generate 2–4 fragments for sequencing equating to approximately 25–50% containing the IS*S1* terminus (matched reads). We identified matched reads at a mean frequency of 38% (range from 28 to 51%; **Table 2**). It may be possible to generate smaller fragments of gDNA in the initial stages of this protocol and these might be expected to yield corresponding shorter inverse PCR products, which would proportionally increase the information content of each PCR product. However, experimentally, following the procedures outlined by Hartl and Ochman (1994), an initial fragment size of ∼3 kb was deemed optimal for generation of a re-circularized inverse PCR template.

Within this study we deemed a gene was essential when no insertion event was detected in the CDS. However, this was expanded to include those CDS where insertion events were detected only beyond the 90th percentile of the sequence. Such CDS may produce an incomplete but sometimes functional protein (carrying a relatively short C-terminal deletion of the gene product). The sequences of the essential and truncated genes detected using PIMMS were compared with other known essential genes obtained from the database of essential genes (DEGs; Zhang et al., 2004; Zhang and Lin, 2009; Luo et al., 2014). In *S. uberis*, genes encoding ribosomal proteins and transfer RNA adaptor molecules dominated the non-mutated (50%) and truncated (30%) CDS. Such sequences are highly conserved across most of the different bacterial species and were also essential in 82% of the genomes contained within the DEG, further indicating the suitability of the PIMMS laboratory protocol and bioinformatic pipeline for detection of essential (and/or conditionally essential) genes. Interestingly, a number of conserved hypothetical sequences within the essential *S. uberis* dataset (sub0149, sub223, sub399, sub1158, sub1413, sub1468, sub1619, sub1832A) were also identified as essential, in other Streptococcal species in the DEG.

Comparison of the ability of CDS associated with glycolysis to tolerate mutation indicated, not unexpectedly, this pathway to be comprised mainly of essential and/or terminally mutated sequences. Where clear redundancy existed, for instance in the case of phosphoglycerate mutase, the orthologous CDS (sub0594; sub0838, sub0839, and sub1509) were all mutated; suggesting that none had functional dominance. The interconversion of glycerate-2P to glycerate-3P may also be effected by a distinct activity, 2,3-di-phosphoglycerate-dependant phosphoglycerate mutase, for which only one ortholog (sub1263; *gpmA*) was detected and this was devoid of mutations indicating its essentiality and/or functional dominance. *S. pyogenes*, also contains multiple sequences encoding both activities capable of interconversion of glycreate-2P and glycerate-3P (Pancholi and Caparon, 2016). An investigation of two strains of *S. pyogenes* (Le Breton et al., 2015) identified three orthologs of gpm. In one strain of *S. pyogenes, gpmA* was essential whilst in another strain none of the sequences was clearly identified as essential. Somewhat surprisingly, and in contrast to the findings of Le Breton et al. (2015), enolase (sub0655) was mutated in our study. Although insertions were not highly prevalent (four unique insertions in the CDS) in the population, indicating a high likelihood of a major role in bacterial fitness, these were not located at the extreme sequence termini implying this activity could be removed (at some fitness cost) under the conditions used. Similarly, detection of a single mutation (present at very low prevalence) in pyruvate kinase around the midpoint of its CDS suggests it also plays a major role in bacterial fitness. Alternative metabolic routes to pyruvate exist via products of the pentose phosphate pathway and from metabolism of acetyl CoA^1^. In addition, other activities encoded within the many hypothetical sequences may play key/redundant roles in metabolism.

The PIMMS pipeline may also be used in line with the annotation independent procedures described by Chao et al. (2016) to examine the role of non-coding regions of DNA. The functional understanding of the roles of non-coding DNA is still in its infancy, however variably sized areas of non-coding DNA fragments are known to bind to transcriptional factors to form enhancer or silencer regulatory regions (van Wolfswinkel and Ketting, 2010). The regions of the *S. uberis* genome that do not code for protein, account for approximately 9% of the total genome sequence. Analysis of these regions revealed a total of 1,149,915 insertion events; 9.8% of all detected insertions. There were 465 intragenic regions where no mutation events could be detected; suggesting some potential functional role may be associated with these sequences, but further detailed analysis is required to substantiate these claims.

Whilst there appears to be new techniques emerging for insertion mutation mapping, the straightforward and pragmatic nature of the laboratory protocol described in this communication: application of a mutagenic technique that is simple, randomly integrating and that does not require a highly transformable host, alongside readily accessible molecular biology techniques and conventional (commercially available) sequencing library preparation and sequencing protocols highlights the PIMMS laboratory methodology as a technology that is very accessible to the wider scientific community to enable functional description and annotation of an increasing list genome-sequenced *Lactococcus, Streptococcus*, and *Enterococcus*.

## Author Contributions

JL and RE conceived the study; SE and AB developed the methodology; AW conducted pathway analysis. JL, AB, RE, and SE wrote the manuscript.

## Conflict of Interest Statement

The authors declare that the research was conducted in the absence of any commercial or financial relationships that could be construed as a potential conflict of interest.

## Footnotes

1 http://www.genome.jp/kegg-bin/show_pathway?sub00010

## Supplementary Material

TABLE S1 **Mapped insertion data for each coding sequence in the genome of *S. uberis* 0140J using PIMMS libraries generated with *HindIII*, *EcoR1*, acoustic shearing (Covaris), no treatment (gDNA); summarized in Table 1.** Mapped insertion data for each coding sequence in the genome of *S. uberis* 0140J using the combined pools of over >80,000 insertions in the final protocol; summarized in **Table 2**.

## References

[B1] Baureder M. Hederstedt L. (2012). Genes important for catalase activity in *Enterococcus faecalis*. *PLoS ONE* 7:e36725 10.1371/journal.pone.0036725 PMC3349705 22590595

[B2] Biswas S. Biswas I. (2011). Role of VltAB, an ABC transporter complex, in viologen tolerance in *Streptococcus mutans*. *Antimicrob.* *Agents Chemother.* 55 1460–1469. 10.1128/AAC.01094-10 PMC3067168 21282456

[B3] Blanchard A. M. Leigh J. A. Egan S. A. Emes R. D. (2015). Transposon insertion mapping with PIMMS – pragmatic insertional mutation mapping system. *Front. Genet.* 6:139 10.3389/fgene.2015.00139 PMC4391243 25914720

[B4] Bradley A. J. Leach K. A. Breen J. E. Green L. E. Green M. J. (2007). Survey of the incidence and aetiology of mastitis on dairy farms in England and Wales. *Vet. Rec.* 160 253–257. 10.1136/vr.160.8.253 17322356

[B5] Chadfield M. S. Christensen J. P. Christensen H. Bisgaard M. (2004). Characterization of streptococci and enterococci associated with septicaemia in broiler parents with a high prevalence of endocarditis. *Avian Pathol.* 33 610–617. 15763731 10.1080/03079450400013089

[B6] Chanter N. (1997). Streptococci and enterococci as animal pathogens. *Soc. Appl. Bacteriol. Symp. Ser.* 26 100S–109S. 10.1046/j.1365-2672.83.s1.11.x 9436322

[B7] Chao M. C. Abel S. Davis B. M. Waldor M. K. (2016). The design and analysis of transposon insertion sequencing experiments. *Nat. Rev. Microbiol.* 14 119–128. 10.1038/nrmicro.2015.7 26775926 PMC5099075

[B8] Chao M. C. Pritchard J. R. Zhang Y. J. Rubin E. J. Livny J. Davis B. M. (2013). High-resolution definition of the *Vibrio cholerae* essential gene set with hidden Markov model-based analyses of transposon-insertion sequencing data. *Nucleic Acids Res.* 41 9033–9048. 10.1093/nar/gkt654 23901011 PMC3799429

[B9] Gawronski J. D. Wong S. M. S. Giannoukos G. Ward D. V. Akerley B. J. (2009). Tracking insertion mutants within libraries by deep sequencing and a genome-wide screen for *Haemophilus* genes required in the lung. *Proc. Natl. Acad. Sci. U.S.A.* 106 16422–16427. 10.1073/pnas.0906627106 19805314 PMC2752563

[B10] Goodman A. L. McNulty N. P. Zhao Y. Leip D. Mitra R. D. Lozupone C. A. (2009). Identifying genetic determinants needed to establish a human gut symbiont in its habitat. *Cell Host Microbe* 6 279–289. 10.1016/j.chom.2009.08.003 19748469 PMC2895552

[B11] Green B. Bouchier C. Fairhead C. Craig N. L. Cormack B. P. (2012). Insertion site preference of Mu, Tn5 and Tn7 transposons. *Mob. DNA* 3:3 10.1186/1759-8753-3-3 PMC3292447 22313799

[B12] Hartl D. L. Ochman H. (1994). Inverse polymerase chain reaction. *Methods Mol. Biol.* 31 187–196. 10.1385/0-89603-258-2:187 7921017

[B13] Hill A. Leigh J. (1989). DNA fingerprinting of *Streptococcus uberis*: a useful tool for epidemiology of bovine mastitis. *Epidemiol. Infect.* 103 165–171. 10.1017/S0950268800030466 2776850 PMC2249475

[B14] Hooven T. A. Catomeris A. J. Akabas L. H. Randis T. M. Maskell D. J. Peters S. E. (2016). The essential genome of *Streptococcus agalactiae*. *BMC Genomics* 17:406 10.1186/s12864-016-2741-z PMC4881062 27229469

[B15] Huang G. Zhang L. Birch R. G. (2000). Rapid amplification and cloning of Tn5 flanking fragments by inverse PCR. *Lett. Appl. Microbiol.* 31 149–153. 10.1046/j.1365-2672.2000.00781.x 10972718

[B16] Hutchison C. A. III Peterson S. N. N. Gill S. R. R. Cline R. T. T. White O. Fraser C. M. M. (1999). Global transposon mutagenesis and a minimal mycoplasma genome. *Science* 286 2165–2169. 10.1126/science.286.5447.2165 10591650

[B17] Krzywinski M. I. Schein J. E. Birol I. Connors J. Gascoyne R. Horsman D. (2009). Circos: an information aesthetic for comparative genomics. *Genome Res.* 19 1639–1645. 10.1101/gr.092759.109 19541911 PMC2752132

[B18] Lampe D. J. Grant T. E. Robertson H. M. (1998) Factors affecting transposition of the *Himar1 mariner* transposon *in vitro*. *Genetics* 149 179–187. 9584095 10.1093/genetics/149.1.179 PMC1460121

[B19] Langridge G. C. Phan M. Turner D. J. Perkins T. T. Parts L. Haase J. (2009). Simultaneous assay of every *Salmonella* Typhi gene using one million transposon mutants. *Genome Res.* 19 2308–2316. 10.1101/gr.097097.109 19826075 PMC2792183

[B20] Le Breton Y. Belew A. T. Valdes K. M. Islam E. Curry P. Tettelin H. (2015). Essential genes in the core genome of the human pathogen *Streptococcus pyogenes*. *Sci. Rep.* 5:9838 10.1038/srep09838 PMC4440532 25996237

[B21] Luo H. Lin Y. Gao F. Zhang C.-T. Zhang R. (2014). DEG 10 an update of the database of essential genes that includes both protein-coding genes and noncoding genomic elements. *Nucleic Acids Res.* 42 574–580. 10.1093/nar/gkt1131 PMC3965060 24243843

[B22] Luo W. Brouwer C. (2013). Pathview: an R/Bioconductor package for pathway-based data integration and visualization. *Bioinformatics* 29 1830–1831. 10.1093/bioinformatics/btt285 23740750 PMC3702256

[B23] Maguin E. Prévost H. Ehrlich S. D. Gruss A. (1996). Efficient insertional mutagenesis in lactococci and other gram-positive bacteria. *J.* *Bacteriol.* 178 931–935. 10.1128/jb.178.3.931-935.1996 PMC177749 8550537

[B24] Martin V. Mohn W. (1999). An alternative inverse PCR (IPCR) method to amplify DNA sequences flanking Tn5 transposon insertions. *J. Microbiol. Methods* 35 163–166. 10.1016/S0167-7012(98)00115-8 10192049

[B25] Murphy E. C. Frick I. M. (2013). Gram-positive anaerobic cocci - commensals and opportunistic pathogens. *FEMS Microbiol. Rev.* 37 520–553. 10.1111/1574-6976.12005 23030831

[B40] Overbeek R. Olson R. Pusch G. D. Olsen G. J. Davis J. J. Disz T. (2014). The SEED and the Rapid Annotation of microbial genomes using Subsystems Technology (RAST). *Nucl. Acids Res.* 42 D206–D214. 10.1093/nar/gkt1226 24293654 PMC3965101

[B26] Pancholi V. Caparon M. (2016). “*Streptococcus pyogenes* Metabolism,” in *Streptococcus pyogenes: Basic Biology to Clinical Manifestations* eds Ferretti J. J. Stevens D. L. Fischetti V. A. (Oklahoma City, OK: University of Oklahoma Health Sciences Center). 26866220

[B27] Pol M. Ruegg P. L. (2007). Relationship between antimicrobial drug usage and antimicrobial susceptibility of gram-positive mastitis pathogens. *J. Dairy Sci.* 90 262–273. 10.3168/jds.S0022-0302(07)72627-9 17183094

[B28] Pritchard J. R. Chao M. C. Abel S. Davis B. M. Baranowski C. Zhang Y. J. (2014). ARTIST: high-resolution genome-wide assessment of fitness using transposon-insertion sequencing. *PLoS Genet.* 10:e1004782 10.1371/journal.pgen.1004782 PMC4222735 25375795

[B29] Salama N. R. Shepherd B. Falkow S. (2004). Global transposon mutagenesis and essential gene analysis of *Helicobacter* pylori. *J. Bacteriol.* 186 7926–7935. 10.1128/JB.186.23.7926-7935.2004 15547264 PMC529078

[B30] Spellerberg B. Pohl B. Haase G. Martin S. Weber-Heynemann J. Lütticken R. (1999). Identification of genetic determinants for the hemolytic activity of *Streptococcus agalactiae* by ISS1 transposition. *J.* *Bacteriol.* 181 3212–3219. 10.1128/jb.181.10.3212-3219.1999 PMC93778 10322024

[B31] Steer A. C. Lamagni T. Curtis N. Carapetis J. R. (2012). Invasive group a streptococcal disease: Epidemiology, pathogenesis and management. *Drugs* 72 1213–1227. 10.2165/11634180-000000000-00000 22686614 PMC7100837

[B32] van Opijnen T. Bodi K. L. Camilli A. (2009). Tn-seq: high-throughput parallel sequencing for fitness and genetic interaction studies in microorganiams. *Nat. Methods* 6 767–772. 10.1038/nmeth.1377 19767758 PMC2957483

[B33] van Wolfswinkel J. C. Ketting R. F. (2010). The role of small non-coding RNAs in genome stability and chromatin organization. *J. Cell Sci.* 123 1825–1839. 10.1242/jcs.061713 20484663

[B34] Ward P. N. Field T. R. Ditcham W. G. F. Maguin E. Leigh J. A. (2001). Identification and disruption of two discrete loci encoding hyaluronic acid capsule biosynthesis genes hasA, hasB, and hasC in *Streptococcus uberis*. *Am. J. Microbiol.* 69 392–399. 10.1128/IAI.69.1.392-399.2001 PMC97895 11119529

[B35] Ward P. N. Holden M. T. G. Leigh J. A. Lennard N. Bignell A. Barron A. (2009). Evidence for niche adaptation in the genome of the bovine pathogen *Streptococcus uberis*. *BMC Genomics* 10:54 10.1186/1471-2164-10-54 PMC2657157 19175920

[B36] Zhang R. Lin Y. (2009). DEG 5.0 a database of essential genes in both prokaryotes and eukaryotes. *Nucleic Acids Res.* 37 455–458. 10.1093/nar/gkn858 PMC2686491 18974178

[B37] Zhang R. Ou H.-Y. Zhang C.-T. (2004). DEG: a database of essential genes. *Nucleic Acids Res.* 32 271–272. 10.1093/nar/gkh024 14681410 PMC308758

[B38] Zomer A. Burghout P. Bootsma H. J. Hermans P. W. M. van Hijum S. A. (2012). ESSENTIALS: software for rapid analysis of high throughput transposon insertion sequencing data. *PLoS ONE* 7:e43012 10.1371/journal.pone.0043012 PMC3416827 22900082

[B39] zur Hausen H. (2006). *Streptococcus bovis*: causal or incidental involvement in cancer of the colon? *Int. J. Cancer* 119 xi–xii. 10.1002/ijc.22314 16947772

